# RNA-combine: a toolkit for comprehensive analyses on transcriptome data from different sequencing platforms

**DOI:** 10.1186/s12859-021-04549-y

**Published:** 2022-01-06

**Authors:** Xuemin Dong, Shanshan Dong, Shengkai Pan, Xiangjiang Zhan

**Affiliations:** 1 Key Laboratory of Animal Ecology and Conservation Biology, Institute of Zoology, Chinese Academy of Sciences, 1 Beichen West Road, Chaoyang District, Beijing, 100101 China; 2 Key Laboratory of Biomedical Information Engineering of Ministry of Education, and Institute of Molecular Genetics, School of Life Science and Technology, Xi’an Jiaotong University, Xi’an, 710049 China; 3 Sino-Danish College, University of Chinese Academy of Sciences, Beijing, 100049 China; 4 CAS Center for Excellence in Animal Evolution and Genetics, Chinese Academy of Sciences, Kunming, 650223 China

**Keywords:** Transcriptome, User-friendly, Visualization, Non-bioinformatician

## Abstract

**Background:**

Understanding the transcriptome has become an essential step towards the full interpretation of the biological function of a cell, a tissue or even an organ. Many tools are available for either processing, analysing transcriptome data, or visualizing analysis results. However, most existing tools are limited to data from a single sequencing platform and only several of them could handle more than one analysis module, which are far from enough to meet the requirements of users, especially those without advanced programming skills. Hence, we still lack an open-source toolkit that enables both bioinformatician and non-bioinformatician users to process and analyze the large transcriptome data from different sequencing platforms and visualize the results.

**Results:**

We present a Linux-based toolkit, RNA-combine, to automatically perform the quality assessment, downstream analysis of the transcriptome data generated from different sequencing platforms, including bulk RNA-seq (Illumina platform), single cell RNA-seq (10x Genomics) and Iso-Seq (PacBio) and visualization of the results. Besides, this toolkit is implemented with at least 10 analysis modules more than other toolkits examined in this study. Source codes of RNA-combine are available on GitHub: https://github.com/dongxuemin666/RNA-combine.

**Conclusion:**

Our results suggest that RNA-combine is a reliable tool for transcriptome data processing and result interpretation for both bioinformaticians and non-bioinformaticians.

## Background

“How can scientists better understand the workings of a cell? Studying the transcriptome, RNA expressed from the genome, reveals a more complex picture of the gene expression behind it all” [1]. In this regard, understanding the transcriptome comprises a prerequisite for full understanding of the biological function of a cell, a tissue and even an organ [1]. Recently, in light of the development of high throughput sequencing technologies (e.g. RNA-seq, Iso-Seq), researchers are able to profile whole transcriptome of an organ, tissue or even a single cell of a species. To mine these data, an increasing number of bioinformatics tools (software) have been developed. Transcriptome analyses involve multiple modules (e.g. expression level calculation, identification of differentially expressed genes, variant calling, co-expression network construction etc.) and the processing of data from different sequencing platforms (e.g. bulk RNA-seq data generated from Illumina platform, scRNA-seq from 10x Genomics platform, Iso-Seq from PacBio platform). Many current tools, however, have been proven both time and labor consuming and not friendly for users, especially non-bioinformaticians, mainly due to the following five issues. First, most existing tools are limited to the data analysis from a single sequencing platform. Second, most tools have only one analysis module. For example, the most recently published tool, RASflow [2], is only designed for the gene expression difference analysis. Third, most tools are developed using different advanced computer languages, (e.g. RNASeqR [3] based on R, scGEAToolbox [4] based on Matlab), which are challenging for non-bioinformatician users. Fourth, the input formats vary among different tools. Lastly, the results generated from most tools can’t be visualized.

Here, we present a Linux-based command-line toolkit, RNA-combine, for the comprehensive analysis of bulk RNA-seq, scRNA-seq as well as Iso-Seq data, performing analyses for multiple modules (e.g. read preprocessing, read alignment, transcript quantification, detection of differential expression and annotation, alternative splicing, result visualization, etc.). This toolkit represents the assembly of a wide range of routine and customized transcriptome analysis workflows and is free from the abovementioned issues and thus is friendly and easily implemented for users.

## Implementation

Our RNA-combine consists of 16 modules and could process transcriptome data (bulk RNA-seq, scRNA-seq, and Iso-Seq) from three sequencing platforms (Illumina, 10x Genomics, PacBio). All analysis modules are scripted in the form of bash command lines, which can be easily customized and launched by users.

**Fig. 1:**
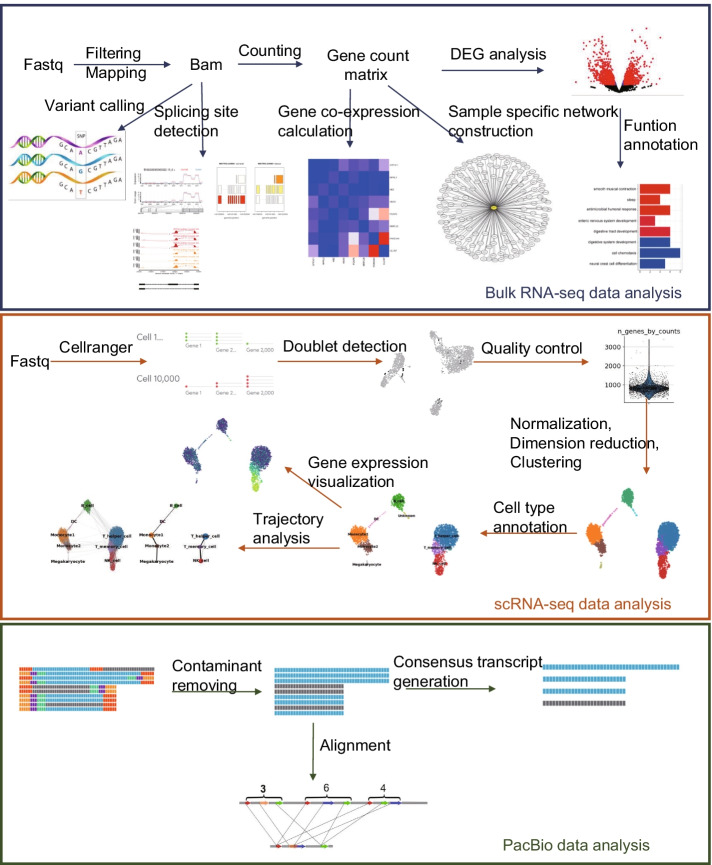
The Schematic workflow of RNA-combine. It includes three analysis units dealing with bulk RNA-seq, scRNA-seq and Iso-Seq data, respectively

The usage of each module is provided in the user manual and the scheme of our toolkit is shown in Fig. 1. Moreover, the organization of all modules and their function descriptions in RNA-combine is shown in Additional file 1: Figure S1.

### Introduction of analysis modules

#### Bulk RNA-seq data analysis

The workflow of bulk RNA-seq data analysis starts with raw sequencing file preprocessing, including removing rRNA sequences using Sortmerna [5], removing adapters and trimming low quality bases using Trimmomatic [6], aligning reads to reference genome using Hisat2 [7], counting reads on genes to calculate gene expression levels using featureCounts [8]. The aligned sequence files and/or gene count matrix can be used as inputs for downstream analysis modules: variant (INDEL and SNP) calling, DEG analysis, function annotation, gene co-expression analysis and splicing site detection.

In the variant calling module, two methods are introduced: the one is conducted by GATK [9], which has been widely used as one of the most reliable methods in the variant identification from RNA-seq data [10]; the other is Strelka2 [11], which shows better sensitivity and precision in variant calling compared with GATK [12]. Notably, we apply three or more methods in each of the three modules: DEG, splicing site detection, gene co-expression analysis. This approach integrates the advantages of different methods. For instance, in DEG module, we use DESeq2 [13], limma [14], edgeR [15] and T-Test which differ from each other in both gene expression normalization and DEG identification: DESeq2 normalizes gene expression with a “geometric” normalization strategy and detects DEGs using an exact test; edgeR uses the weighted mean of log ratios for normalization and an exact test for DEG identification; limma adopts a quantile normalization approach and an empirical Bayesian analysis for DEG detection, whereas T-Test method applies a T-Test on *RPKM* (*Reads Per Kilobase of transcript per Million mapped reads*)/*FPKM* (*Fragments Per Kilobase of exon model per Million mapped fragments*) normalized data. In the module of splicing site detection, three methods with different strategies are adopted: DEXseq [16] based on exon boundaries, StringTie [17] based on transcripts, and rMATS [18] based on splicing events. In the module of gene co-expression, we use linear-association based Pearson and Spearman coefficients, both of which have been widely used in constructing gene co-expression networks. We also apply a recently published method PMI [19], which measures nonlinearly direct dependencies based on the part mutual information among variables (gene expression). We further adopt SSN [20], which can construct a sample-specific network relying on the gene expression profiles of control samples and process one case sample. We introduce clusterProfiler [21] to the functional enrichment (GO pathways and KEGG pathways) module. It is noted that with the aligned sequence files and gene count matrix, users can customize workflows to meet specific analysis requirements.

#### scRNA-seq data analysis

Cellranger [22] is used for preprocessing raw fastq files, including the read alignment and generation of feature-barcode matrices. The feature-barcode matrices can be passed onto downstream analyses.

Currently, the library construction used for scRNA-seq often causes doublet artifacts (i.e. two cells have the same barcode, thus mistakenly regarded as one cell), which may generate biased results. To overcome this limitation, we apply Scrublet [23] in the toolkit, which can identify such artifacts and exclude them before further analyses. Then, we utilize SCANPY [24] to conduct a sequence of processing and analysis, including the quality control, dimension reduction, cell clustering, cell type annotation, trajectory inference, providing users with the visualization of the number of genes in cells, percentage of mitochondrial genes in cells, cell cluster atlases, cell connectivity, and cell trajectories. To assign each cell cluster to a cell type, we develop a new method, based on two classical cell marker databases, CellMarker [25] and PanglaoDB [26]. Taken the DEG list of a cell cluster of interest (against with other cell clusters) as the input, our method searches related cell type information for each DEG in the two databases, with the cell type showing the largest hit number considered as the true one.

#### Iso-Seq data analysis

We use one of the most well-established pipeline-PacBio’s IsoSeq V3 [27] to process raw sequencing data, including polishing, hierarchical clustering, iterative merging to obtain consensus full-length transcripts. For the read alignment, we suggest users to use local alignment methods (e.g. Minimap2 [28] in our toolkit), since the length of Iso-Seq reads is much longer than those generated by RNA-seq.

### Interface of the input format

For omics (e.g. transcriptome) analysis, one of the most labor-intensive and time-consuming processes is the formatting of input files for different tools. In our toolkit, we develop a layer of interface that could automatically format inputs for user-specified tools, guaranteeing that these tools could be linked smoothly. Under this environment, bioinformaticians and non-bioinformaticians could quickly link different tools to a reliable processing workflow without extra commands. More importantly, the users just need to input the workflow with raw transcriptome sequencing data and reference genomes. Our toolkit will start from initial preprocessing and load the modules of interest. For instance, in the analysis of bulk RNA-seq data, one can set up a workflow starting from raw sequencing data preprocessing, to the calculation of expression level of each gene or transcript, identification of differentially expressed gene or transcript, detection of alternative splicing event and differential splicing site, construction of co-expression network and sample-specific network, variant calling (SNPs, INDELs), and biological pathway annotation.

### Visualization

Because each module of transcriptomic analysis involves thousands of genes and multiple dimensions (e.g. genes, networks etc.), the data presentation with graphical visualization is in need to facilitate the result interpretation. However, this has been neglected in most of the previous tools. To address this issue, we develop a visualization plugin under the environment of R and Python, which realizes the visualization of the results for each module and can generate qualified graphs for publication.

### Backtracking and recording

During the processing of large datasets (e.g. transcriptomes of multiple samples), spurious interruption might occur due to unexpected factors (e.g. interruption of power supply, storage overload). This abolishes the entire workflow and users have to spend a lot efforts to manually check all the processes. To minimize this effect as much as possible, we develop a Backtracking Executor plugin in our toolkit. It automatically records the interrupted processes (i.e. error logs) in a user-specified directory. Based on these log files, it is easy for users to resume data processing from the interrupted points. As a consequence, the plugin enables users to keep tracking the processes and thus greatly reducing the efforts spent on debugging.

## Results and discussion

### Case studies

**Fig. 2:**
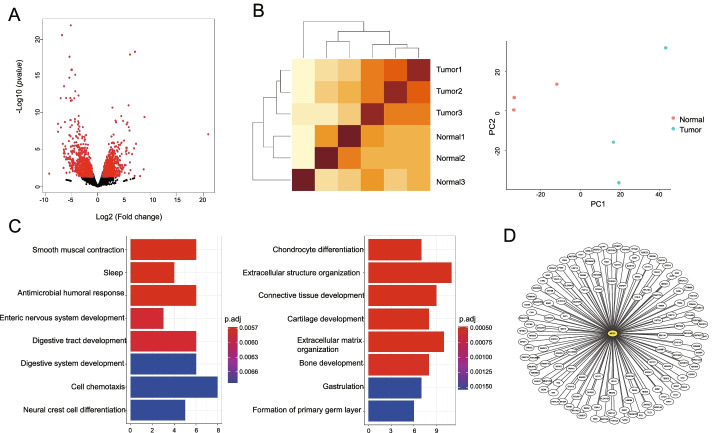
Application of bulk RNA-seq data analysis workflow to breast tumor datasets. **a** Volcano plot of DEGs between breast tumor and normal breast samples. **b** Heatmap and PCA (principal component analysis) plots of sample distances. **c** Functional pathway enrichment of DEGs in normal (left) and tumor (right) samples. **d** Differentially co-expressed network between tumor and normal samples with *MUC1* as a hub

**Fig. 3:**
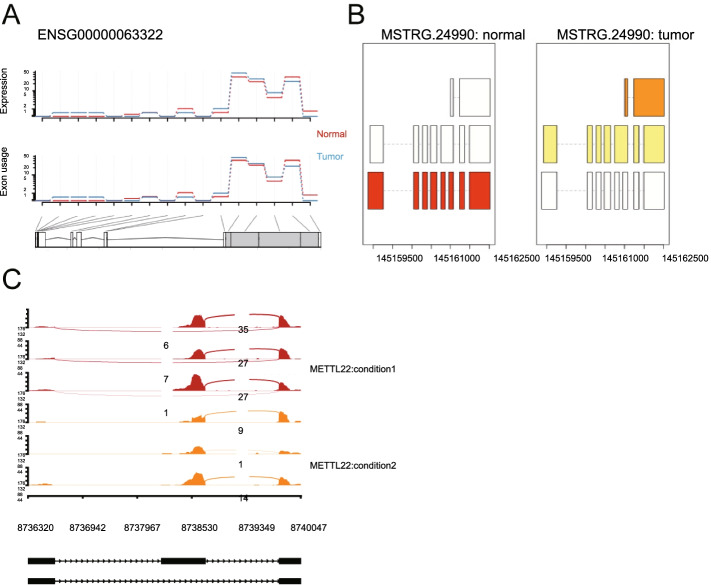
Examples of differential splicing sites in tumor and normal conditions identified from exon-based (**a**), transcript-based (**b**), event-based (**c**) approaches

In this section, we focused on the assessments of reliability and automation of RNA-combine using several published transcriptome data. We first tested the workflow of the bulk RNA-seq analysis with a transcriptome dataset from three normal breast tissues and three breast tumor tissues [29]. Starting from the fastq files, RNA-combine first removed contaminated sequences, aligned sequences to reference genome, counted read numbers on genes, producing a gene count matrix, which was then passed for the differential expression analysis, co-expression analysis and splicing site detection. The results of differentially expressed genes between tumor and normal conditions and the enriched functional terms were shown in Fig. 2a, c, and the sample distances were shown in Fig. 2b. Among the identified differentially expressed genes, *MUC1*, which has been approved by FDA as a diagnostic marker to monitor clinical course of patients with breast tumor during treatment [30], was significantly upregulated in the tumor samples, suggesting that our toolkit was reliable. Genes specifically co-expressed with *MUC1* in either tumor or normal tissues were shown in Fig. 2d. Moreover, three genes with differential splicing sites (exon-based, transcript-based and event-based) identified between tumor and normal samples were shown in Fig. 3.

**Fig. 4:**
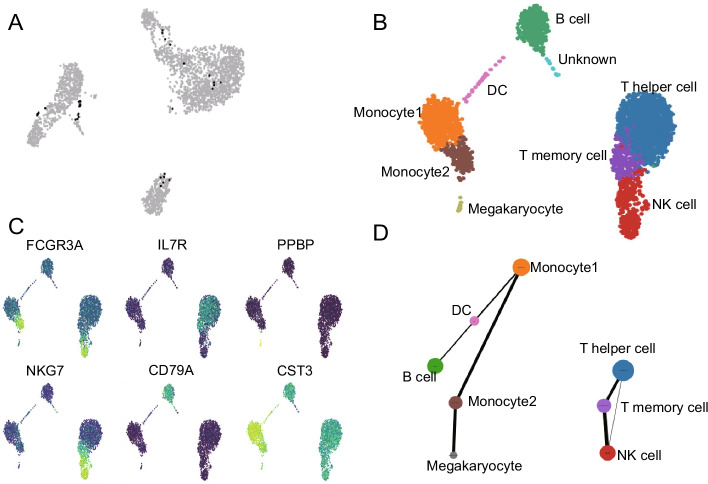
Application of scRNA-seq data analysis workflow to 3k PBMC scRNA dataset. **a** UMAP (Uniform Manifold Approximation and Projection) plot of predicted single cells (grey) and doublets (black). **b** UMAP plot of 9 cell clusters. **c** Examples of expression of classical cell markers, *FCGR3A* is a marker for FCGR3A+ monocytes, *IL7R* for T cells, *PPBP* for megakaryocytes, *NKG7* for natural killer cells, *CD79A* for B cells, and *CST3* for monocytes and monocyte derived dendritic cells. **d** Cell connectivity of cell clusters

Next, using the pipeline of scRNA-seq data analysis in RNA-combine we analyzed a scRNA-seq dataset of 3k PBMCs (peripheral blood mononuclear cell) from a healthy donor generated from 10x Genomics (https://support.10xgenomics.com). The gene-barcode matrix was used for doublet detection, cell clustering, cell type annotation and cell connectivity analysis. The number of 35 candidates were detected as potential doublets among 2700 cells (Fig. 4a) and thus were discarded. The clustering results were shown in Fig. 4b. These cell clusters were further annotated according to classical cell markers, such as *NKG7* for natural killer cells and T cells [31], *PPBP* for Megakaryocytes [32], *CD79A* for B cells [33] (Fig. 4c). The cell connectivity analysis revealed two main branches (lymphocytes and myeloid cells), as shown in Fig. 4d.

We used the public Alzheimer’s Iso-Seq dataset (1 percentage of total samples) [34] to test the pipeline of Iso-Seq. This test produces a total of 2245 high-quality full-length transcripts. Estimated running time of each module in all of the above analyses is shown in Additional file 1: Table S1.

Overall, these results suggested that our toolkit worked smoothly and automatically for real transcriptome data from different sequencing platforms and it could process the data with raw sequencing fastq files and reference genomes as inputs.

### Compared with other methods

**Fig. 5:**
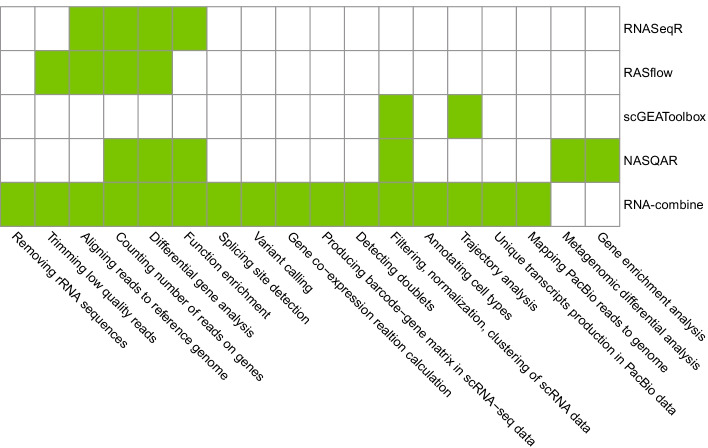
Functions in RNA-combine and other four toolkits

We compared the features of RNA-combine with four toolkits (RNASeqR, RASflow, scGEAToolbox, NASQAR [35]) published in recent 2 years (Fig. 5). Among them, RNASeqR and RASflow could only process bulk RNA-seq data, and scGEAToolbox is designed for scRNA-seq data only. Although NASQAR can process both bulk RNA-seq and scRNA-seq data, it can’t process Iso-Seq data.

Regarding the analysis module, our toolkit covers all the analysis modules implemented in RNASeqR, RASflow, scGEAToolbox, and most modules in NASQAR (except for metagenomic differential analysis and gene enrichment analysis), and it also includes nine extra modules (i.e. rRNA removal, alternative splicing site detection, variant detection, gene-co-expression network construction, scRNA gene-barcode matrix production, doublet detection, cell type annotation, PacBio sequence alignment and full-length transcript analysis) that are absent in the abovementioned software. One major difference between RNA-combine and other toolkits is that it implements the modules of raw data preprocessing, which enables users to use raw fastq files as input directly. Another difference is the development of the input formatting interface, which automatically formats inputs for different analysis modules, therefore enabling end-to-end analysis from raw sequencing files to direct graphic visualization of results. This is important because input formatting is both time- and labor-consuming, especially for users without advanced programming skills. RNA-combine is a Linux-based toolkit, enabling users to parallel jobs, thus speeding up the analysis processes. Collectively, RNA-combine is more comprehensive on data analysis and more user-friendly.

## Conclusions

We conclude that RNA-combine presented in this study is a user-friendly, reliable toolkit for the comprehensive analysis of transcriptome data generated from different sequencing platforms. It enables users to set up a customized workflow to analyze data from multiple platforms and generate analysis reports and result visualization. Importantly, this toolkit empowers researchers without advanced bioinformatics skills to analyze their data by working with human-readable configuration files. We will continue to provide user supports and feature enhancements in future releases.

## Availability and requirements

- Project name: RNA-combine
- Project home page: The software and usage guideline are available online at https://github.com/dongxuemin666/RNA-combine
- Operating system(s): Linux
- Programming language: Python, R, Shell
- Other requirements: R 3.6, Python 3.7
- License: MIT, licenses for dependent packages, tools and methods are shown in Additional file 1: Table S2
- Any restrictions to use by non-academics: license needed

## Additional file

**Additional file 1.** Estimated running time for each module, organization of all modules and functions, licenses for dependent packages of RNA-combine.

## Data Availability

Bulk RNA-seq data analysis: six samples (SRR868857, SRR868862, SRR868865, SRR868869, SRR868873, SRR868877) from NCBI Sequence Read Archive. scRNA-seq data analysis: scRNA data of 3k PBMCs from a healthy donor provided by 10x (http://cf.10xgenomics.com/samples/cell-exp/1.1.0/pbmc3k). Iso-Seq data analysis: from the website https://downloads.pacbcloud.com/public/dataset.

## Abbreviations

scRNA-seq: Single cell RNA sequencing
SNP: Single nucleotide polymorphism
INDEL: Insertion or deletion of bases
rRNA: Ribosomal RNA
DEG: Differentially expressed gene
GO: Gene Ontology
KEGG: Kyoto Encyclopedia of Genes and Genomes
SSN: Sample-specific network
FDA: Food and Drug Administration
