# Supplementary material for: RNA-combine: a toolkit for comprehensive analyses on transcriptome data from different sequencing platforms
Source: BMC Bioinformatics. 2022 Jan 6;23:26. doi: 10.1186/s12859-021-04549-y (PMC8740077; doi:10.1186/s12859-021-04549-y)
Supplement: Supplementary file 1 — Additional file 1. Estimated running time for each module, organization of all modules and functions, licenses for dependent packages of RNA-combine. [file 12859_2021_4549_MOESM1_ESM.pdf]

1 Estimated running time for each module

The tests were conducted on three different servers: Server 1 (CPU: Intel(R) Xeon(R) Gold 6230 CPU @ 2.10GHz, 160 processors; Memory: 2014 G); Server 2 (CPU: Intel(R) Xeon(R) CPU E5-2620 v4 @ 2.10GHz, 16 processors; Memory: 62 G); and Server 3 (CPU: Intel(R) Xeon(R) Gold 5218 CPU @ 2.30GHz, 32 processors; Memory: 125G). For each server, only one thread is used (except for "Bulk RNA-seq pre-processing" module).

Table S1. Estimated running time for each module

Module	Time cost	Data (Input)	Server
Bulk RNA-seq pre-processing	5h 7m 20s (20 threads)	6 fastq files (24.4G)	3
Variant detection (GATK)	34m 13s	3 BAM files (1.1G), chromosome 1	3
Mutation detection (Strelka2)	8m 23s	2 SAM files (7.8G), chromosome 1	2
Differential analysis (DESeq2)	46s	1 gene expression matrix (6 samples, 62492 transcripts)	2
Differential analysis (edgeR)	13s	1 gene expression matrix (6 samples, 62492 transcripts)	2
Differential analysis (limma)	8s	1 gene expression matrix (6 samples, 62492 transcripts)	2
Differential analysis (T-test)	29s	1 gene expression matrix (6 samples, 62492 transcripts)	2
Sample-specific network construction	22h 3m 26s	Gene expression matrices (9 samples, 15371 transcripts)	1
Gene function enrichment (GO)	1m 19s	1 gene list with 40 genes	2

Gene function enrichment (KEGG)	12s	1 gene list with 40 genes	2
Gene co-expression network construction (PCA-PMI)	2.662s	1 gene expression matrix (6 samples, 37 transcripts)	2
Gene co-expression network construction (Pearson)	0.836s	1 gene expression matrix (6 samples, 37 transcripts)	2
Gene co-expression network construction (Spearman)	0.826s	1 gene expression matrix (6 samples, 37 transcripts)	2
Splicing site detection (DEXseq)	1h 9s 34s	6 SAM files (18.3G)	2
Splicing site detection (StringTie+ballgown)	26m 5s	6 BAM files (3.1G)	2
Splicing site detection (rMATS)	4m 40s	6 BAM files (3.1G)	2
CellRanger	1h 17m 54s	1 sample with 3 fastq files (21.9G)	1
Doublet detection	5m 12s	1 sparse gene-barcode matrix (183M)	2
Cell clustering	2m 41s	1 gene-barcode matrix (671M)	2
Cell type searching	5.239s	1 gene list with 29 genes	2
Labeling cell types	5.319s	1 h5ad object (22M)	2
Gene expression visualization	7.115s	1 h5ad object (22M)	2
Trajectory analysis	8.589s	1 h5ad object (22M)	2

Producing consensus transcripts using Iso-seq3	8m 27s	1 BAM file, 5.8G	3
Alignment using Minimap2	3m 34s	1 BAM file, 76M<br>1 reference genome, 3G	2

Data sources:

Bulk RNA-seq data analysis: six samples (SRR868857, SRR868862, SRR868865, SRR868869, SRR868873, SRR868877) from the project of Brunner et al. [1].

scRNA-seq data analysis: SRR5799774 sample from the project of Savage et al. [2].

Iso-Seq analysis: data from the website:

https://downloads.pacbcloud.com/public/dataset/IsoSeq_sandbox/2020_Alzheimer8M_subset/alz.1perc.subreads.bam.

2 The organization of all modules and their functions in RNA-combine

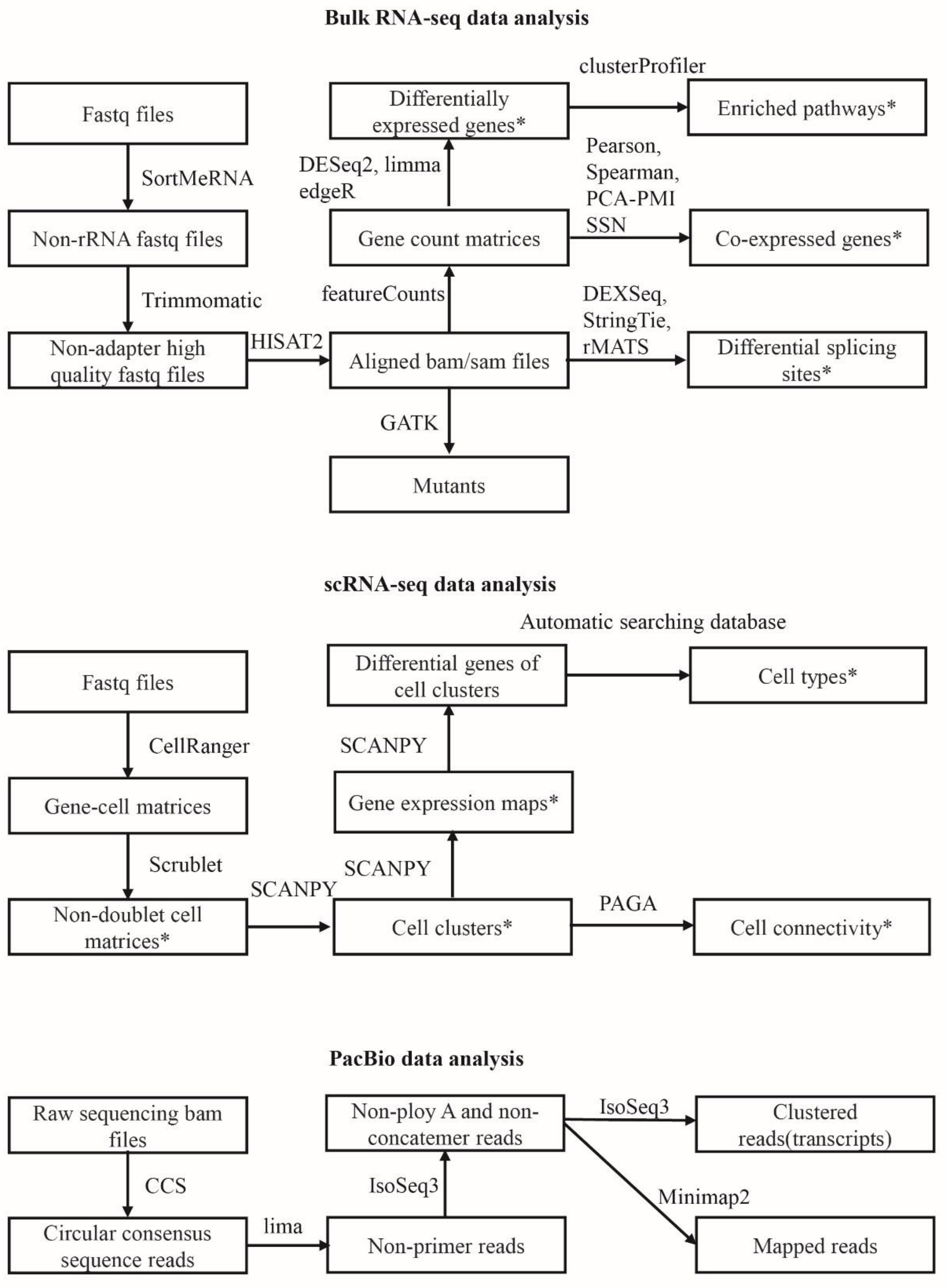

Figure S1. Organization of all modules and their functions in RNA-combine. * Results could be visualized.

3 Licenses for dependent packages, tools and methods

Table S2. Licenses for dependent packages, tools and methods

Tool	License
SortMeRNA	LGPL
Trimmomatic	GPLv3
DESeq2	LGPL (≥3)
limma	GPL (≥2)
edgeR	GPL (≥2)
featureCounts	GPLv3
GATK	BSD-3-Clause
clusterProfiler	Artistic-2.0
DEXSeq	GPL (≥3)
StringTie	MIT
rMATS	Free for non-commercial use
CellRanger	https://github.com/10XGenomics/cellranger/blob/master/LICENSE
Scrublet	MIT
SCANPY	BSD-3
CCS	BSD-3-Clause-Clear
IsoSeq3	BSD-3-Clause-Clear
Minimap2	MIT

References

[1] Brunner AL, Li J, Guo X, Sweeney RT, Varma S, Zhu SX, et al. A shared transcriptional program in early breast neoplasias despite genetic and clinical distinctions. Genome Biol. 2014;15(5):R71.

[2] Savage P, Blanchet-Cohen A, Revil T, Badescu D, Saleh SMI, Wang YC, et al. A Targetable EGFR-Dependent Tumor-Initiating Program in Breast Cancer. Cell Rep. 2017;21(5):1140-1149.
